# Editorial: Precision psychiatry from a pharmacogenetics perspective

**DOI:** 10.3389/fpsyt.2023.1159000

**Published:** 2023-03-02

**Authors:** Lisa C. Brown, Josiah D. Allen, Harris A. Eyre, Bernhard T. Baune, Katherine J. Aitchison, Chad A. Bousman

**Affiliations:** ^1^Great Scott! Consulting LLC, New York, NY, United States; ^2^Medigenics Consulting LLC, Cincinnati, OH, United States; ^3^St. Elizabeth Healthcare, Edgewood, KY, United States; ^4^Center for Health and Bioscience, The Baker Institute for Public Policy, Rice University, Houston, TX, United States; ^5^Meadows Mental Health Policy Institute, Dallas, TX, United States; ^6^Department of Psychiatry, University of Münster, Münster, Germany; ^7^Department of Psychiatry, The University of Melbourne, Parkville, VIC, Australia; ^8^Department of Psychiatry, College of Health Sciences, University of Alberta, Edmonton, AB, Canada; ^9^Department of Medical Genetics, College of Health Sciences, University of Alberta, Edmonton, AB, Canada; ^10^Department of Neuroscience and Mental Health Institute, College of Health Sciences, University of Alberta, Edmonton, AB, Canada; ^11^Women and Children's Health Research Institute, College of Health Sciences, University of Alberta, Edmonton, AB, Canada; ^12^Northern Ontario School of Medicine, Sudbury, ON, Canada; ^13^Department of Medical Genetics, University of Calgary, Calgary, AB, Canada

**Keywords:** pharmacogenetics, pharmacogenomic (PGx) research, psychiatry, genetics, precision medicine, personalized medicine

The field of psychiatric pharmacogenomics has undergone a period of turbulence in recent years. The United States Food and Drug Administration (FDA) began taking action on laboratory testing claims in November 2018, with a specific focus on laboratories making claims with respect to psychiatric medication efficacy and tolerability. In their communications on the subject, the FDA made clear that they found evidence for clinical utility of psychiatric pharmacogenomic testing to be lacking and instructed laboratories to limit information shared on their test products. This action resulted in the removal of drug-gene interpretation reports and language from a number of commercial test offerings, potentially significantly reducing their clinical utility. This action was met with resistance by a number of groups including the National Alliance for Mental Illness (NAMI), the Depression and Bipolar Support Alliance (DBSA), the Mental Health Alliance (MHA), the Clinical Pharmacogenetics Implementation Consortium (CPIC), the Association for Molecular Pathology (AMP), and the Coalition to Preserve Access to Pharmacogenomics.

Meanwhile, United Healthcare (UHC), the largest commercial payor in the United States, released a policy that expanded coverage of pharmacogenomic testing for individuals with major depressive and anxiety disorders. At the same time, Palmetto GBA, the Medicare Administrative Contractor (MAC) in charge of the MolDx molecular diagnostic review process, released a new Local Coverage Determination expanding the scope of covered pharmacogenomic testing through Medicare, both in terms of covered conditions, covered genes, and prescribing clinicians. The apparent divergence between FDA regulatory activity and increased willingness on the part of payors to reimburse for psychiatric pharmacogenomic testing underscores the need for additional quality research in the field.

This Special Issue of Precision psychiatry from a pharmacogenetics perspective includes nine articles highlighting the current and emerging evidence for psychiatric pharmacogenomics as well as clinical implementation considerations. This special issue is a valuable overview of the current state of pharmacogenomics in psychiatry and how it can be used to personalize treatment for individuals with mental illness.

## Current evidence

Beatriz et al. provide a comprehensive introduction to metabolism in psychiatric pharmacogenomics (Henriques et al.). From discussion of the CYP system and specific medications to future work in the field. The authors provide a concise overview of relevant enzymes to psychiatric medications and the most recent data supporting their use in guiding medication treatment. van Westrhenen et al. builds on Beatriz et al. by reviewing clinical pharmacogenomics and the authors conclude that the most clinically relevant pharmacogenomic information is connected to pharmacokinetic drug metabolism, specifically, CYP2C19 and CYP2D6 genetic variations. In the application of pharmacogenomics, Aldrich et al., leaders in the field of pharmacogenomics in child and adolescent psychiatry, conducted a retrospective EMR review study to understand the potential role of CYP2C19 in response and side effects in adolescents with anxiety. Specifically, they found a significant increase in activation in adolescents who were poor metabolizers for CYP2C19. For ultrarapid metabolizers, the authors found that patients responded faster. This study is an important step in understanding the value of pharmacogenomics in guiding dosing for children and adolescents to decrease side effects and improve outcomes. Beyond just gene-drug interactions, Cook et al. investigated the effect of antidepressant treatment on peripheral blood gene expression in patients with MDD. They found differential expression between treatment with escitalopram and bupropion. Notably, they also found overlap of 18 genes implicated in a previous study differentiating patients with MDD from positive controls. The evidence for pharmacodynamic genes affect outcomes in psychiatry is lacking compared to the pharmacokinetic evidence (Li et al.). However, in this study by Li et al., found that variations in the HTR2C gene resulted in differential response to antipsychotics in patients with schizophrenia. This study adds to previous literature linking HTR2C with response as well as risk of weight gain with antipsychotics.

## Implementation considerations

Education and implementation are crucial pieces to successfully utilizing pharmacogenomic testing, Maggo et al. found that in patients with severe adverse reactions to psychotropic treatment and subsequent referral for pharmacogenomic testing are enriched with genetic variations in CYP2D6 and CYP2C19. This study shows the value of pharmacist utilization of pharmacogenomic testing to investigate abnormal reactions and side effects to psychotropic medications. One caveat to pharmacogenomic testing is the effect of ancestry on genotype frequencies and outcomes. Cui et al. sought to determine specific dosing recommendations of risperidone for individuals of Asian and Caucasian descent with variation in CYP2D6 metabolism. Specifically, the authors found that doses of risperidone should be reduced by 45% in Caucasians and 26% for Asians who are poor metabolizers at CYP2D6. They also found that doses should be increased by 30 and 33% in Asians and Caucasians, respectively. Overall, the authors estimate that different ethnicities with the same phenotypes may require differing dosages of risperidone. Additionally, many pharmacogenomic genes are incredible polymorphic. Jarvis et al. discuss the complexity of CYP2D6 variation and copy number variants in interpreting and applying this information to pharmacogenomics in psychiatry. They discuss the intricacy around the sheer number of variants of 2D6, detection of these variants, and interpretation and application. It's common knowledge that variants in HLA genes increasing risk of adverse skin reactions to carbamazepine are more common in Asian ancestry (Fang et al.). However, this study by Moreno et al. found that only screening patients with Asian ancestry could be missing a large portion of the population at risk. These data support the use of pharmacogenomic HLA testing before starting carbamazepine regardless of ethnic background to avoid severe, and sometimes deadly, skin adverse reactions.

## Future directions

Pharmacogenomic testing has made significant progress in the last 4 years, including additions to the current evidence base, emerging evidence, and implementation.

The future of pharmacogenomics may include incorporation of clinical features along with genetics and, combined with the burgeoning fields of machine learning and artificial intelligence techniques, may further personalize medication decisions for individual patients. Finally, we look forward to seeing the rise of polygenic risk scores to medication response and are optimistic about seeing clinical utility studies in this space in the next few years.

## Author contributions

LB and JA wrote initial draft. CB, HE, BB, and KA edited final publication. All authors contributed to the article and approved the submitted version.

